# Adsorption of β-galactosidase of *Alicyclobacillus acidocaldarius* on wild type and mutants spores of *Bacillus subtilis*

**DOI:** 10.1186/1475-2859-11-100

**Published:** 2012-08-03

**Authors:** Teja Sirec, Andrea Strazzulli, Rachele Isticato, Maurilio De Felice, Marco Moracci, Ezio Ricca

**Affiliations:** 1 Department of Structural and Functional Biology, Federico II University of Naples, Naples, Italy; 2 Institute of Protein Biochemistry, C.N.R., Naples, Italy

## Abstract

**Background:**

The *Bacillus subtilis* spore has long been used as a surface display system with potential applications in a variety of fields ranging from mucosal vaccine delivery, bioremediation and biocatalyst development. More recently, a non-recombinant approach of spore display has been proposed and heterologous proteins adsorbed on the spore surface. We used the well-characterized β-galactosidase from the thermoacidophilic bacterium *Alicyclobacillus acidocaldarius* as a model to study enzyme adsorption, to analyze whether and how spore-adsorption affects the properties of the enzyme and to improve the efficiency of the process.

**Results:**

We report that purified **β-**galactosidase molecules were adsorbed to purified spores of a wild type strain of *B. subtilis* retaining ca. 50% of their enzymatic activity. Optimal pH and temperature of the enzyme were not altered by the presence of the spore, that protected the adsorbed **β-**galactosidase from exposure to acidic pH conditions. A collection of mutant strains of *B. subtilis* lacking a single or several spore coat proteins was compared to the isogenic parental strain for the adsorption efficiency. Mutants with an altered outermost spore layer (crust) were able to adsorb 60-80% of the enzyme, while mutants with a severely altered or totally lacking outer coat adsorbed 100% of the **β-**galactosidase molecules present in the adsorption reaction.

**Conclusion:**

Our results indicate that the spore surface structures, the crust and the outer coat layer, have an negative effect on the adhesion of the **β-**galactosidase. Electrostatic forces, previously suggested as main determinants of spore adsorption, do not seem to play an essential role in the spore-**β-**galactosidase interaction. The analysis of mutants with altered spore surface has shown that the process of spore adsorption can be improved and has suggested that such improvement has to be based on a better understanding of the spore surface structure. Although the molecular details of spore adsorption have not been fully elucidated, the efficiency of the process and the pH-stability of the adsorbed molecules, together with the well documented robustness and safety of spores of *B. subtilis*, propose the spore as a novel, non-recombinant system for enzyme display.

## Background

Display systems to present biologically active molecules on the surface of microorganisms have become an increasingly used strategy to address biotechnological issues [1,2]. For biomedical applications surface display systems have been mostly used for the identification of neutralizing epitopes, the development of whole cell diagnostic tools, or vaccine delivery [3,4]. More recent is a strategy to engineer bacterial endospores (spores) to display heterologous proteins on their surface [5]. Endospore-forming bacteria are Gram-positive microorganisms belonging to different genera and including more than 1,000 species [6]. The common feature of these organisms is the ability to form a quiescent cellular type (the spore) in response to harsh environments. The spore can survive in this dormant state for long periods, resisting to a vast range of stresses such as high temperature, dehydration, absence of nutrients, presence of toxic chemicals. When the environmental conditions ameliorate, the spore germinates originating a vegetative cell able to grow and sporulate [6]. The ability of the spore to survive non-physiological conditions is, in part, due to the presence of the spore coat, a proteinaceous structure surrounding the spore. At least seventy different proteins (Cot proteins) form the multilayered coat structure, composed of an inner part, an outer part [7] and the crust, the latter being a recently discovered outermost layer of the spore [8,9].

Spore-based display systems provide several advantages with respect to systems based on the use of phages and bacterial cells [10]. The remarkable and well documented resistance of spores to various environmental and toxic effects [7] ensures high stability of the display system. Proteins to be displayed on the spore are produced in the mother cell compartment of the sporangium and are assembled around the forming spore without the need to be translocated across a membrane, thus eliminating the size constrains of cell-based display systems [5,10]. The safety record of several endospore-forming species [11], makes spores of those species ideal candidates as vehicles to deliver molecules to mucosal surfaces [6].

The strategy to obtain the spore surface display of heterologous proteins is based on the construction of gene fusions between the gene coding for a selected spore surface protein (carrier) and the heterologous DNA coding for the protein to be displayed [5]. By this approach a variety of heterologous proteins have been displayed and recombinant spores proposed as vaccine vehicles (see ref. 6 for a review), as biocatalysts (see ref. 10 for a review), or as a bioremediation tool [12]. To optimize and rationalize this display strategy an inner (OxdD [13]) and various outer (CotB [5], CotC [14,15], CotG [16]) coat components have been tested as carriers.

The spore-based display system, like other cell- or phage-based systems, relies on the genetic engineering of the host to display immunogenic peptides or proteins and obtain a recombinant organism to be used as a live biotechnological tool [5,6,10]. This is a major drawback since it causes the release of live recombinant organisms into nature, raising concerns over the use and clearance of genetically modified microorganisms [17]. To overcome this obstacle, a non-recombinant approach to use spores as a display system has been recently proposed and model proteins efficiently exposed. In the first study suggesting that heterologous proteins can be adsorbed on the spore surface, the mammalian NADPH-cytochrome P450 reductase (CPR), a diflavin-containing enzyme, was over-expressed in sporulating *B. subtilis* cells and released into the culture medium after sporulation by autolysis of the mother cell. However, part of the CPR activity was found associated to spores and the displayed enzyme shown to be accessible to anti-CPR antibodies [18]. In a different study a collections of purified antigens (TTFC of *Clostridium tetani*, PA of *Bacillus anthracis*, Cpa of *Clostridium perfringens* and glutathione S transferase of *Shistosomas japonica*) were adsorbed to *B. subtilis* spores and shown to be able to induce specific and protective immune responses in mucosally immunised mice [19]. Spore adsorption resulted to be more efficient when the pH of the binding buffer was acidic (pH 4) and less efficient or totally inhibited at pH values of 7 or 10 [19]. A combination of electrostatic and hydrophobic interactions between spores and antigens were suggested to drive the adsorption, that was shown to be not dependent on specific spore coat components but rather on the negatively charged and hydrophobic surface of the spore [19]. Hydrophobic and electrostatic interactions were suggested as the main forces involved also in the interaction between the *E. coli* phytase and spores of *B. polyfermenticus*[20].

We used a well-characterized and biotechnologically important enzyme, a β-galactosidase of the thermoacidophilic bacterium *Alicyclobacillus acidocaldarius*[21], as a model to study enzyme adsorption on *B. subtilis* spores*.* This enzyme belongs to the glycoside hydrolase family 42 (GH42) and is characterized by an optimal activity and stability at 65°C [21]. By using this system we tested whether adsorbed β-galactosidase molecules retained their activity and whether and how spore-adsorption affected the properties of the enzyme. With the dual aim of identifying spore surface structures involved in β-galactosidase adsorption and to improve the efficiency of the process we also screened for enzyme binding a collection of mutant strains of *B. subtilis* lacking a single or several spore coat proteins. A better understanding of the spore surface structure is likely to lead to a rationalization of the adsorption system, such that wild type or mutant spores will be utilized, depending upon the specific application or the heterologous enzyme to display.

## Results

### β-Galactosidase of *A. acidocaldarius* adsorbs to *B. subtilis* spores and retains its enzymatic activity

**Figure 1:**
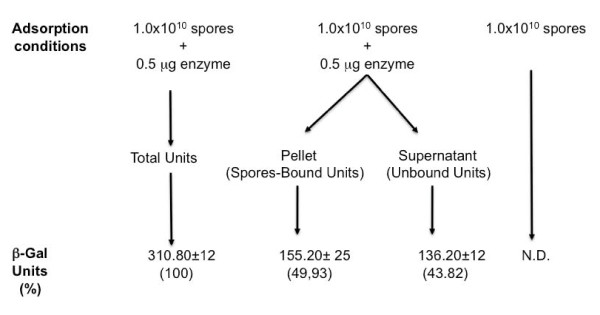
**Schematic representation of a typical adsorption experiment.** Purified spores were mixed to the purified enzyme in PBS buffer (pH 4.0) and incubated one hour at 25°C. The reaction was then stopped in ice and sample either directly assayed (Total Units) or fractionated by centrifugation and fractions assayed independently.

In an initial experiment 0.5 μg of β-Galactosidase (β-Gal) of *A. acidocaldarius*, over-expressed in *E. coli* and purified by affinity chromatography with GST columns (Methods), were incubated with 1.0×10^10^ spores of *B. subtilis* strain PY79 [22], purified by renographin gradient, as previously described [23]. The adsorption reaction was performed in citrate buffer at pH 4.0, as previously described for various antigens [19]. After adsorption, the mixture was either assayed for β-Gal activity ("Total Units" in Figure1) or fractionated by centrifugation. The pellet ("Spore-Bound Units" in Figure1) and supernatant ("Unbound Units" in Figure1) fractions were then assayed independently. Figure1 schematizes the experiment and reports that ca. 50% of the β-Gal units were found in the pellet fraction and, therefore, were most likely associated to the spores. The spore-associated β-Gal activity was not due to an endogenous enzyme, since purified spores alone did not show any activity (Figure1). Only a limited amount (less than 10%) of β-Gal units was lost during the procedure (Figure1) and the adhesion appeared to be stable, since 95% of the units was still associated to the pellet fraction after two washes with phosphate buffer pH 4.0 or 5.5 (not shown).

In a previous report Huang et al. [19] have shown that spore adsorption of four different antigens is more efficient at pH 4.0 than at pH 7.0 or 10.0. In agreement with those observations we found that adsorption of β-Gal to 1.0×10^10^ spores was strictly dependent on the pH of the adsorption buffer, with a similar amount of units adsorbed at pH values of 3.5 and 4.0 and a strongly decreased amount of units adsorbed at pH 4.5 (Table1). β-Gal has a deduced isoelectric point of 5.77, therefore at all pH tested in the experiments of Table1, it is expected to have a net positive charge. At pH values lower than 3.5 no activity was found in any of the fractions as well as in the unfractionated sample (Total Units), indicating that exposure at pH 3.0 totally inactivated the enzyme (Table1). At both pH values of 3.5 and 4.0 the amount of β-Gal units lost in the experimental procedure was about 10% (Table1).

**Table 1:** **pH-dependent adsorption efficiency with 1 × 10**^**10**^**spores**

**pH values of the adsorption reaction**				
	**3.0**	**3.5**	**4.0**	**4.5**
**Total units (%)**	0	262.55 ± 5.15 (100)	308.92 ± 11.13 (100)	353.64 ± 23.80 (100)
**Spore-bound units (%)**	0	139.64 ± 5.65 (53.18)	158.98 ± 2.41 (51.46)	26.89 ± 4.68 (7.60)
**Unbound-units (%)**	0	92.46 ± 8.24 (35.21)	127.77 ± 12.38 (41.36)	306.72 ± 26.96 (86.73)

^1^β-galactosidase unit is defined as an amount of β-Gal which is able to hydrolyze 1 μmol of substrate in 1 min at standard conditions [19].

**Figure 2:**
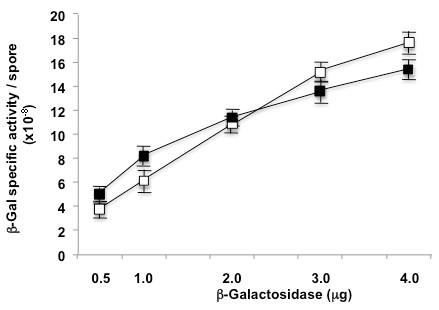
**β-galactosidase units per spore obtained with increasing amounts of enzyme.** The enzyme was adsorbed to 2.0×10^9^ (open symbols) or 1.0×10^10^ (closed symbols) spores.

To check that our experimental conditions were not limiting for the number of spores, we measured the β-Gal activity associated to each spore using different amounts of enzyme and spores in the adsorption reaction. As shown in Figure2, with both 2.0×10^9^ (open symbols) and 1.0×10^10^ (closed symbols), increasing the amount of enzyme used in the adsorption reaction up to 4 μg, the β-Gal units bound to each spore increased, indicating that the spores present in the reaction buffer were not saturated by the enzyme and therefore were not a limiting factor for the reaction.

### Adsorption to spores stabilizes β-Gal

**Figure 3:**
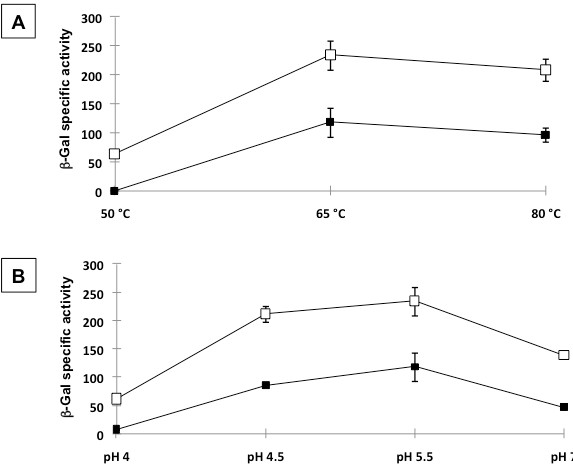
Spore-associated β-galactosidase units obtained assaying the samples at pH 5.5 and various temperatures (A) or at 65°C and various pH values (B).

It has been previously reported that the β-Gal of *A. acidocaldarius* has an optimal activity and stability at 65°C and at pH 6.5 [21]. To verify whether spore adsorption affected these properties, we compared the β-Gal activity of the free and the spore-adsorbed enzyme, after exposing both to the adsorption conditions (1 hour at pH 4.0). As shown in Figure3, the optimum temperature (panel A) and pH (panel B) were identical with (open symbols) and without (closed symbols) spores, indicating that the spore-bound and the free enzyme have identical properties.

**Figure 4:**
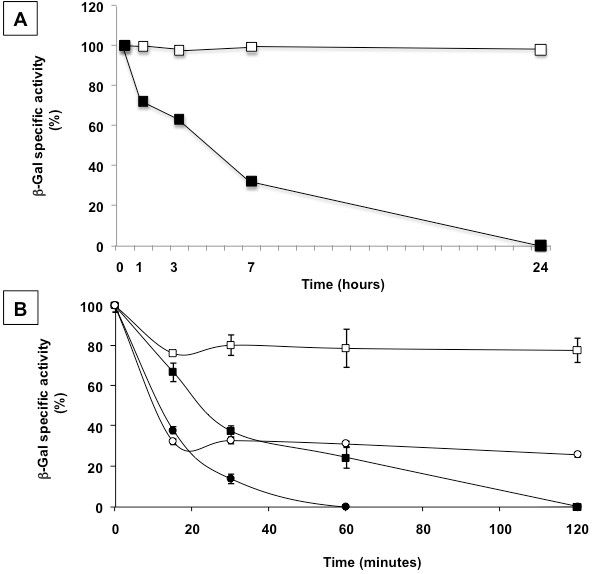
**(A) Percentage of β-galactosidase units observed after incubation at pH 4.0 for various times of free (closed squares) and spore-bound (open squares) enzyme.** (**B**) Percentage of β-galactosidase units observed after incubation at 75°C (squares) and 80°C (circles) for various times of free (closed symbols) and spore-bound (open symbols) enzyme. The data shown are representative of three independent experiments, each with three biological replicates.

Results of Figure3 indicate that at all tested temperatures and pH values the spore-bound enzyme is more active than the free enzyme. A stabilization effect of spores on an enzyme has been previously suggested for a different enzyme and for spores of a different bacterial species [20]. To address this point in more detail we compared the activity of spore-bound and free β-Gal after exposure at low pH or high temperatures. Although the enzyme is from a thermoacidophilic bacterium, the activity of the free enzyme decreased overtime after exposure at both low pH or high temperature and was protected by denaturation when adsorbed to spores. After exposure at pH 4.0 the activity of the free β-Gal was completely lost after 24 hours while the spore-bound enzyme was still fully active (Figure4A). After 2 hours of incubation at 75°C the activity of the free β-Gal was completely lost while the spore-bound enzyme retained about 80% of its original activity (black and white squares in Figure4B, respectively). At 80°C the activity of the free enzyme was completely lost after 1 hour while the spore-bound enzyme retained over 30% of its activity even after two hours of incubation (black and white circles in Figure4B, respectively).

### Spores with altered surface show increased efficiency of adsorption

**Figure 5:**
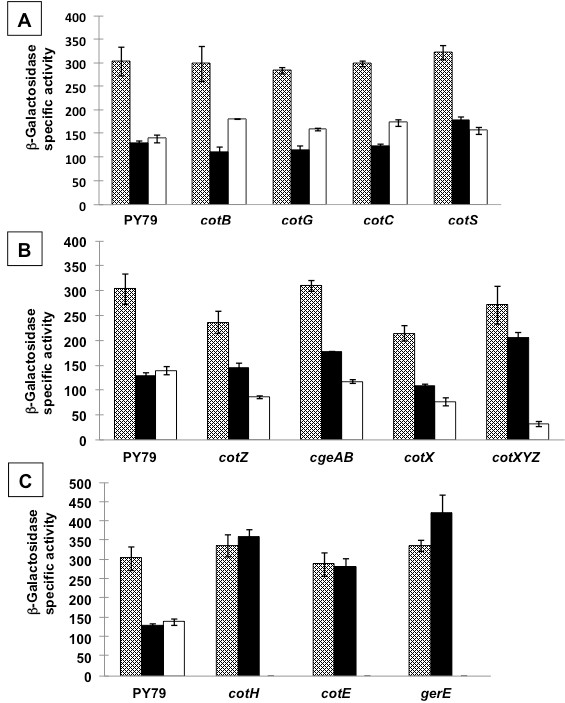
**Total Units (grey bars), Spore-Bound Units (black bars) and Unbound Units (white bars) of β-galactosidase obtained using wild type (PY79) or isogenic mutant spores.** Panel **A** includes mutants lacking a single outer coat component; panel **B** includes mutants totally or partially lacking the crust; and panel **C** includes mutants with a severely defective (*cotH* and *gerE*) or totally absent (*cotE*) outer coat.

In a previous report it has been shown that adsorption of various antigens on the surface of *B. subtilis* spores does not depend on a direct interaction with any of the major outer coat components (CotA, CotB, CotC, CotG and CotF) [19]. In agreement with the previous results we showed that β-Gal adsorption is similar in wild type spores (PY79) and in isogenic null mutants lacking *cotB, cotC, cotG* or *cotS* (Figure5A). However, it has been recently shown that the *B. subtilis* spore is surrounded by an outermost layer, the crust, not identified before [8]. The crust is composed of proteins and the products of the *cgeA* gene and of the *cotVWXYZ* cluster are involved in crust formation [9]. One of those proteins, CotZ, has been proposed to be essential for the spore crust to surround the spore but not for the formation of the other coat layers [9]. Based on this, we examined whether the presence of the crust had an effect on the adsorption of β-Gal by comparing the efficiency of adsorption of a wild type strain (PY79) with that of isogenic null mutants lacking *cotZcgeA cotX* or *cotXYZ*. As shown in Figure5B, the efficiency of β-Gal adsorption was increased in all four mutants. Only a slight increase was observed with mutant spores lacking *cotZ, cgeA* or *cotX* (from ca. 50% of activity associated to wild type spores to ca. 60% associated to the mutant spores), while a stronger effect was observed with spores lacking *cotXYZ* (ca. 80% of spore-associated activity). Since the mutant lacking *cotXYZ* showed a higher increase of β-Gal adhesion than mutants lacking only *cotZ* or *cotX,* we hypothesize that either CotY is an important factor in mediating the adhesion of β-Gal or that CotZ, CotX and CotY have an additive action in modifying the spore surface and, as a consequence, reducing adhesion of the enzyme (Figure5B). Although further experiments will be needed to fully address this point, the results of Figure5B indicate that a normally formed crust has an inhibiting role on β-Gal adhesion.

Next, we decided to test the adsorption efficiency of mutants lacking also other coat layers. In particular, we used spores of strains lacking *cotH, gerE* or *cotE. cotH* spores lack at least 9 outer coat components [24], *gerE* codes for a transcriptional regulator and *gerE* mutant spores lack the latest class of coat components [7], and *cotE* spores totally lack the outer coat [25]. As shown in Figure5C, with spores of all three mutants 100% of the β-Gal activity was adsorbed, indicating that the spores lacking the crust and at least part of the outer coat are extremely efficient in adsorbing β-Gal.

### Adsorption to mutant spores lacking *cotE* or *cotH*

Wild type spores of *B. subtilis* are negatively charged [26] and able to accumulate a large number of protons in an aqueous solution [27]. The presence of a large number of spores is then likely to alter the pH of an aqueous solution. Because of the pH-dependence of β-Gal adsorption (Table1), we hypothesized that wild type and mutant spores had a different effect on the pH of the binding solution and, as a consequence, on the efficiency of β-Gal adsorption. To verify this hypothesis we measured the effects of spores on the pH of pure water and of the adsorption buffer (citrate buffer pH 4.0). We tested *cotE* or *cotH* mutant spores lacking either the entire outer coat or 9 major outer coat components [24], while we did not consider *gerE* mutant spores, although they also showed 100% of β-Gal adhesion (Figure5C), since the outer coat structure of *gerE* spores has not been studied in details and not all genes controlled by the transcriptional regulator GerE have been identified and functionally characterized. As shown in Table2, both 2.0×10^9^ and 1.0×10^10^ wild type spores were able to reduce the pH of pure water of over one pH unit, while the same numbers of *cotE* or *cotH* spores had a minor, if any, effect. In all cases no effects on the pH were observed at the adsorption conditions, that buffered the effect of spores (not shown). Those results seem to suggest that the different efficiency of adsorption between wild type and mutant spores was not caused by a spore-dependent alteration of the pH in the adsorption reaction and that wild type spores have a net negative charge higher than that of both *cotE* or *cotH* mutant spores.

**Table 2:** Effect of wild type and mutant spores on pH

	**n° of spores**^**a**^	**pH**^**b**^
Water	0	5.98 ± 0.08
Water + WT spores	2.0 × 10^9^	7.23 ± 0.07
	1.0 × 10^10^	7.14 ± 0.24
Water + *cotE* spores	2.0 × 10^9^	6.06 ± 0.07
	1.0 × 10^10^	6.18 ± 0.04
Water + *cotH* spores	2.0 × 10^9^	5.88 ± 0.07
	1.0 × 10^10^	5.85 ± 0.07

^a^ The number of purified spores was measured by direct counting with a Burker chamber under an optical microscope (Olympus BH-2 with 40xlenses); ^b^ pH was measured using a pH-meter with a combined glass microelectrode (LLBiotrode, Metrohm) probe in a final volume of 200 μL.

**Figure 6:**
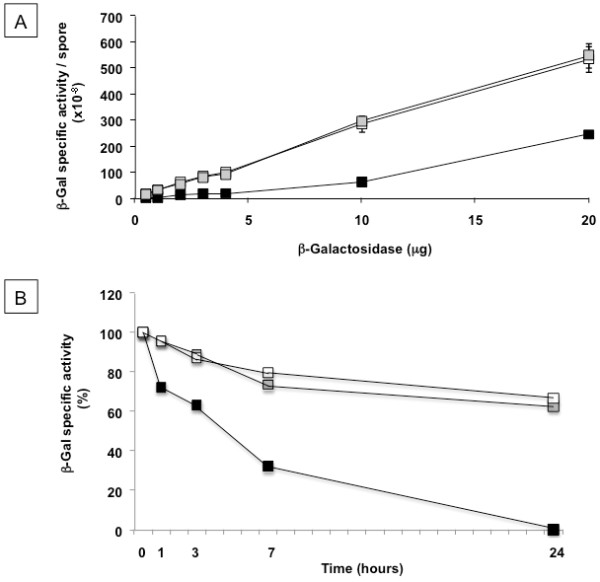
**(A) β-galactosidase units per spore obtained with increasing amounts of enzyme.** The enzyme was adsorbed to wild type spores (black symbols) or mutant spores lacking *cotE* (grey symbols) or *cotH* (white symbols). (**B**) Percentage of β-galactosidase units observed after incubation at pH 4.0 for various times of the free enzyme (black symbols) and of the enzyme bound to *cotE* (grey symbols) or *cotH* (white symbols) spores. The data shown are representative of three independent experiments, each with three biological replicates.

Wild type and mutant spores were then compared for their efficiency in adsorbing and stabilizing β-Gal molecules. *cotE* and *cotH* mutant spores showed identical adsorption efficiencies that increased with increasing amounts of enzyme in the adsorption reaction (Figure6A). These results confirm and expand the results of Figure2, indicating that the spores present in the reaction buffer (2.0×10^9^) were not saturated by 20 μg of enzyme and therefore were not a limiting factor for the reaction. β-Gal has a molecular mass of 77,737 daltons, therefore the maximal amount of enzyme used in the experiment of Figure6A (20 μg) contains approximately 1.55×10^14^ molecules. Since in the adsorbing reaction with 1.0×10^10^ spores about 50% of the enzyme is bound to wild type spores and 100% to mutant spores, we can calculate that about 7.75×10^3^ and 1.55×10^4^ molecules of β-Gal were adsorbed to each wild type and mutant spores, respectively.

**Figure 7:**
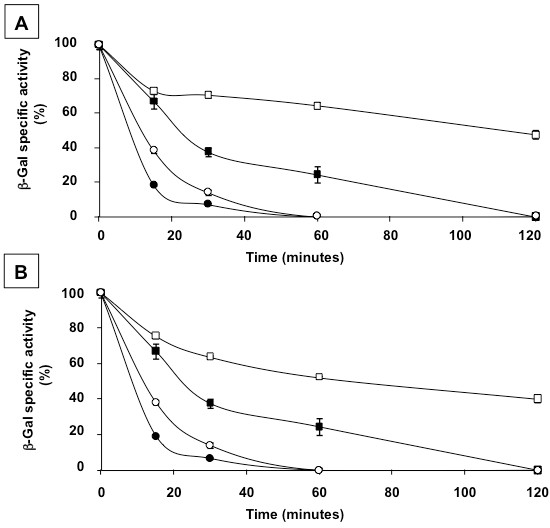
**Percentage of β-galactosidase units observed after incubation at 75°C (squares) and 80°C (circles) for various times of free enzyme (closed symbols) and of the enzyme bound to *cotE* (A) or *cotH* (B) spores (open symbols).** The data shown are representative of three independent experiments, each with three biological replicates.

We then decided to check whether also *cotE* and *cotH* spores, like their isogenic parental strain, were able to stabilize the enzyme at an acidic condition or at high temperatures able to fully denaturate the free enzyme (Figure4). The enzyme bound to either *cotE* or *cotH* spores retained over 70% of its initial activity after 24 hours at pH 4.0, whereas the free enzyme was totally inactive (Figure6B). The enzyme bound to mutant spores lacking either *cotE* (Figure7A) or *cotH* (Figure7B) retained over 50% of their original activity after 2 hours of incubation at 75°C while the free enzyme was totally denatured. At 80°C only a minor protective effect was shown by both *cotE* or *cotH* mutant spores (Figure7). While β-Gal bound to wild type spores was fully active after 24 hours at pH 4.0 and retained about 80 and 40% of its activity after 2 hours at 75°C or 80°C, respectively (Figure4), adsorption to *cotE* or *cotH* spores resulted in reduced stabilization effect to both acidic pH (Figure6B) and high temperatures (Figure7). However, considering that mutant spores adsorb a higher amount of β-Gal activity than wild type spores (Figures 5C and 6A), even with a loss of activity during incubation at denaturing conditions, the use of *cotE* or *cotH* spores can be considered advantageous for specific applications that involve exposure of the enzyme at low pH conditions or high temperatures.

## Discussion

The adsorption of antigens and enzymes to bacterial spores has been reported previously and the involvement of a combination of physicochemical forces has been suggested [18-20]. In this frame, our work was aimed at gaining a better understanding of the spore-enzyme interaction. We used spores of a laboratory strain of *B. subtilis*, for which a collection of isogenic mutants altered in spore surface proteins was available, and as a model enzyme the well characterized β-Gal of the thermoacidophilic bacterium *Alicyclobacillus acidocaldarius*[21].

Our results indicate that spores bind β-Gal without affecting its properties but, instead, stabilizing it at acidic pH and high temperatures. Spore adsorption is a very efficient process with each wild type spore able to adsorb about 7.75×10^3^ molecules of β-Gal. Gene fusion-based display of heterologous antigens was slightly less efficient than adsorption, with 1.5×10^3^ molecules of TTFC (5) and 4.8×10^3^ molecules of LTB (15) exposed on each spore. Spores lacking the outermost structures, the crust and the outer coat layer, have an adsorption efficiency even higher than wild type spores, with 1.55×10^4^ molecules of β-Gal adsorbed on each spore. This indicates that those structures, mainly formed by proteins and glycoproteins [7], have an inhibitory effect on the adhesion of the enzyme. While the efficiency of adhesion is improved, the stabilization at low pH is reduced in the mutants after 24 hours of incubation at pH 4.0. In the case of β-Gal, the loss of activity (30%) at low pH is compensated by the high amount of enzyme adsorbed. Although other enzymes have not been tested yet, our data suggest that the use of wild type or mutants spores can be planned according to the specific application and the heterologous enzyme to be displayed.

The carboxyl groups were identified as the major ionizable groups in the spore and proton diffusion was found much lower in the spore core than within the coats and cortex, suggesting the inner membrane, separating core from the external layers of the spore, as a major permeability barrier for protons [27]. Then, the carboxyl groups in the coat and in the cortex have been suggested as responsible of the negative charge of spores [27]. The different effects of wild type and mutant spores on the pH of an aqueous solution (Table2), indicate that wild type spores attract more protons than *cotE* or *cotH* mutant spores and therefore, suggest that thay have a net negative charge higher than that of the mutant spores. Since spores of those mutants lack either the entire outer coat (*cotE*) or a large part of it (*cotH*), they have a reduced number of proteins, and as a consequence of carboxyl groups, in their cortex and coat. Based on this we hypothesize that the reduced number of carboxyl groups present in the regions of the spore more permeable to proton diffusion [27] is responsible of the reduced number of protons attracted by mutant spores. However, the different efficiency of β-Gal adsorption observed between wild type and mutant spores is not due to a different effect on the pH, since the effect of spores on the pH is buffered at the adsorption conditions (citrate buffer pH 4.0).

Our results, indicating that spores with altered surface structures have altered adsorption efficiency, point to the physicochemical properties of the spore surface as responsible of the interaction with the model enzyme. β-Gal, having a deduced isoelectric point of 5.77, at the adsorption conditions (pH 4.0) is expected to have a net positive charge and to be attracted by negatively charged spores. In spite of this, β-Gal binds more efficiently to mutant (lower negative charge) than wild type (higher negative charge) spores. We conclude that, at least in the case of β-Gal, the electrostatic force does not seem to be the predominant force involved in the interaction with the spore.

The hypothesis that the different negative charge of wild type and mutant spores is somehow responsible of the different stabilization effect observed at pH 4.0 is an intriguing possibility that needs to be addressed. Answering the questions of what is the basis of spore adsorption and of enzyme stabilization are challenging future goals that will necessarily require further experiments and the use of sophisticated physicochemical tools.

Although the molecular details of adsorption and enzyme stabilization have not been totally elucidated, spores have shown clear potentials as a novel, surface display system that, being non-recombinant, able to protect the heterologous enzyme from acidic pH and based on a host with a remarkable safety record [10,11], appear particularly well suited for the delivery of biotherapeutic molecules to animal and human mucosal surfaces.

## Methods

### Bacterial strains and transformation

*B. subtilis* strains used in this study are listed in Table3. *B. subtilis* PY79 [21] was used as recipient strain in transformation procedures. Plasmid amplification for DNA sequencing, subcloning experiments, and transformation of *E. coli* competent cells were performed with strain DH5α [28]. Bacterial strains were transformed by previously described procedures, i.e., CaCl_2_-mediated transformation of *E. coli* competent cells [28] and two-step transformation of *B. subtilis*[23] (Table4).

**Table 3:** ** *B. subtilis* strains**

**Strain**	**Genotype**	**Source**
PY79	Wild type	[22]
RH201	*cotB::spc*	[5]
RH101	*cotC::spc*	[34]
ER203	*cotG::Δerm*	[35]
AZ541	*cotS::cm*	This study
GC347	*cgeA::spc*	This study
AZ542	*cotZ::neo*	This study
AZ543	*cotX::neo*	This study
AZ544	*cotXYZ::neo*	This study
DZ213	*cotE::cm*	[25]
ER220	*cotH::spc*	[36]
KS450	*gerE36*	[35]

**Table 4:** Synthetic oligonucleotides

**Oligonucleotide**	**Sequence (5’- 3’)**^**a**^	**Restriction site**	**Position of annealing**^**b**^
cotS3	ggatccATCGACCATGTGGCGCTG	*Bam*HI	+64/+82
cotS4	aagcttAACCATCACTTTATTCAG	*Hind*III	+771/+758
cotZ1	gcatgcGCTGTTGAAGAAGACTGC	*Sph*I	+64/81
cotZ2	gtcgacAACTTCAATACAGTAGTTCG	*Sal*I	+378/+359
cgeA1	gcatgcCAACTGCAACAGAAGGAG	*Sph*I	+64/+81
cgeA2	gtcgacGTGAACCTGATCGAAAGC	*Sal*I	+297/+279

^a^ Capital and lowercase letters indicate nucleotides complementary to corresponding gene DNA and unpaired flanking sequences carrying a restriction site, respectively. ^b^ Referred to *cotS*, *cotZ* or *cgeA* sequences, taking the first nucleotide of the initiation codon as +1.

### Genetic and molecular procedures

Isolation of plasmids, restriction digestion, and ligation of DNA, were carried out by standard methods [28]. Chromosomal DNA from *B. subtilis* was isolated as described elsewhere [29].

For the construction of insertional *null* mutations DNA fragments internal to genes *cotS, cotZ* and *cgeA* were amplified by PCR using the *B. subtilis* chromosome as a substrate and synthetic oligonucleotides listed in Table2 to prime the reactions. The PCR products were visualized on ethidium bromide-stained agarose gels and gel purified by the QIAquick gel extraction kit (Qiagen) as specified by the manufacturer. Amplified fragments were ligated into plasmids pBEST501 [30] (*cotZ* mutant) or pER19 [31] (*cotS* mutant), carrying an antibiotic resistance cassette selectable in *B. subtilis*. The mutant in *cgeAB* was obtained by cloning amplified internal fragment of *cgeA* gene in a pGEM-T (Promega) vector in which was previously cloned a spectinomycin cassette (from pAH256 [32]) in a *Pst*I restriction site. Those recombinant plasmids were used to transform competent cells of strain PY79. Transformants were the result of a single (Campbell-like) recombination event between homologous DNA present on the plasmid and on the chromosome. Transformants were selected by antibiotic resistance and confirmed by PCR analysis of chromosomal DNA. Mutants in the *cotX* and *cotXYZ* genes were already available but carried in a *B. subtilis* strain with a different genetic background (strain MB24) [33]. To obtain *cotX* and *cotXYZ* mutants isogenic with the wild type and other mutants used in this study, chromosomal DNA of the existing strains was extracted and used to transform competent cells of PY79 competent cells.

### Purification of spores and β-galactosidase

Sporulation of wild type and recombinant strains was induced by the exhaustion method. After 30 h of growth in Difco Sporulation medium (DSM) at 37°C with vigorous shaking [29], spores were collected, washed three times with distilled water and purified by gastrografin gradient as described before [29]. Spore counts were determined by serial dilution and plate-counting.

A recombinant plasmid containing the *lacB* gene of *Alyciclobacillus acidocaldarius* into the expression vector pET29a has been previously described [20]. Expression of *lacB* was induced by 0.1 mM isopropyl-β D-thiogalactoside (IPTG) in *E. coli* BL21RB791 cells and β-Gal purified using the GST-tag and the thrombin cleavage on the matrix as described by the manufacturer (Amersham Biotech).

### Binding assay and enzyme detection

Purified β-Gal was added to a suspension of 1×10^10^ spores in sodium citrate 50 mM at pH 4.0 at 25°C in a final volume of 200 μl. After 1 hour of incubation, an aliquot (70 μl) of the binding mixture was stored at 4°C while the remaining part of the binding mixture was centrifuged (10 min at 13,000 rpm) to fractionate pellet and supernatant. All fractions were then used for β-Gal assays: 20 μl of each fraction were added to the reaction buffer (50 mM sodium citrate buffer at pH 5.5, 2NP-β-D-Gal 10 mM) and mixtures incubated at 65°C for 5 minutes; the reaction was then blocked by addition of 800 μl of 1 M Na_2_CO_3_. When the assay was performed on samples containing spores, the samples were centrifuged prior to measurement of optical density at 420 nm. We expressed results of enzymatic assays in total units, where 1 unit is defined as an amount of β-Gal able to hydrolyze 1 μmol of substrate in 1 min at standard conditions [20].

## Competing interests

The authors declare that they have no competing interests.

## Authors’ contributions

TS performed most of the experiments. AS contributed to enzyme purification and enzymatic assays. RI contributed to the construction of mutants and to develop the adsorption conditions. MDF contributed to experiment design and manuscript writing. MM contributed to experiment design and manuscript writing. ER contributed discussions and suggestions during the work and wrote most of the manuscript. All authors read and approved the final manuscript.

## References

[B1] Wu CH Mulchandani A Chen W Versatile microbial surface-display for enviromental remediation and biofuels production Trends Microbiol 2008 16 181 188 10.1016/j.tim.2008.01.003 18321708

[B2] Lee SY Choi JH Xu Z Microbial cell-surface display Trends Biotechnol 2003 21 45 52 10.1016/S0167-7799(02)00006-9 12480350

[B3] Wells J Mucosal vaccination and therapy with genetically modified lactic acid bacteria Annu Rev Food Sci Technol 2011 2 423 445 10.1146/annurev-food-022510-133640 22129390

[B4] Villaverde A Nanotechnology, biotechnology and microbial cell factories Microb Cell Fact 2010 9 53 10.1186/1475-2859-9-53 20602780 PMC2916890

[B5] Isticato R Cangiano G Tran HT Ciabattini A Medaglini D Oggioni MR De Felice M Pozzi G Ricca E Surface display of recombinant proteins on Bacillus subtilis spores J Bacteriol 2001 183 6294 6301 10.1128/JB.183.21.6294-6301.2001 11591673 PMC100119

[B6] Cutting SM Hong HA Baccigalupi L Ricca E Oral Vaccine Delivery by Recombinant Spore Probiotics Int Rev Immunol 2009 28 487 505 10.3109/08830180903215605 19954360

[B7] Henriques AO Moran CP Structure, assembly and function of the spore surface layers Ann Rev Microbiol 2007 61 555 588 10.1146/annurev.micro.61.080706.093224 18035610

[B8] McKenney PT Driks A Eskandarian HA Grabowski P Guberman J Wang KH Gitai Z Eichenberger P A distance-weighted interaction map reveals a previously uncharacterized layer of the Bacillus subtilis spore coat Curr Biol 2010 20 934 938 10.1016/j.cub.2010.03.060 20451384 PMC2920530

[B9] Imamura D Kuwana R Takamatsu H Watabe K Proteins involved in formation of the outermost layer of Bacillus subtilis spores J Bacteriol 2011 193 4075 4080 10.1128/JB.05310-11 21665972 PMC3147665

[B10] Knecht LD Pasini P Daunert S Bacterial spores as platforms for bioanalytical and biomedical applications Anal Bioanal Chem 2011 400 977 989 10.1007/s00216-011-4835-4 21380604

[B11] Cutting SM Bacillus probiotics Food Microbiol 2011 28 214 220 10.1016/j.fm.2010.03.007 21315976

[B12] Hinc K Ghandili S Karbalaee G Shali A Noghabi K Ricca E Ahmadian G Efficient binding of nickel ions to recombinant Bacillus subtilis spores Res Microbiol 2010 161 757 764 10.1016/j.resmic.2010.07.008 20863881

[B13] Potot S Serra CR Henriques AO Schyns G Display of recombinant proteins on Bacillus subtilis spores, using a coat-associated enzyme as the carrier Appl Environ Microbiol 2010 76 5926 5933 10.1128/AEM.01103-10 20601499 PMC2935070

[B14] Mauriello EMF Duc LH Isticato R Cangiano G Hong HA De Felice M Ricca E Cutting SM Display of heterologous antigens on the Bacillus subtilis spore coat using CotC as a fusion partner Vaccine 2004 22 1177 1187 10.1016/j.vaccine.2003.09.031 15003646

[B15] Isticato R Scotto Di Mase D Mauriello EMF De Felice M Ricca E Amino terminal fusion of heterologous proteins to CotC increases display efficiencies in the Bacillus subtilis spore system Biotechniques 2007 42 151 156 10.2144/000112329 17373477

[B16] Hinc K Isticato R Dembek M Karczewska J Iwanicki A Peszyńska-Sularz G De Felice M Obukowski M Ricca E Expression and display of UreA of Helicobacter acinonychis on the surface of Bacillus subtilis spores Microb Cell Fact 2010 9 2 10.1186/1475-2859-9-2 20082702 PMC2841587

[B17] Detmer A Glenting J Live bacterial vaccines—a review and identification of potential hazards Microb Cell Fact 2006 5 23 10.1186/1475-2859-5-23 16796731 PMC1538998

[B18] Yim S-K Jung H-C Yun C-H Pan J-G Functional expression in Bacillus subtilis of mammalian NADPH-cytochrome P450 oxidoreductase and its spore-display Protein Expr Purif 2009 63 5 11 10.1016/j.pep.2008.07.004 18678259

[B19] Huang JM Hong HA Van Tong H Hoang TH Brisson A Cutting SM Mucosal delivery of antigens using adsorption to bacterial spores Vaccine 2010 28 1021 1030 10.1016/j.vaccine.2009.10.127 19914191

[B20] Cho EA Kim EJ Pan JG Adsorption immobilization of Escherichia coli phytase on probiotic Bacillus polyfermenticus spores Enzyme Microb Technol 2011 49 66 71 10.1016/j.enzmictec.2011.03.006 22112273

[B21] Di Lauro B Strazzulli A Perugino G La Cara F Bedini E Corsaro MM Rossi M Moracci M Isolation and characterization of a new family 42 beta-galactosidase from the thermoacidophilic bacterium Alicyclobacillus acidocaldarius: identification of the active site residues Biochim Biophys Acta 2008 1784 292 301 10.1016/j.bbapap.2007.10.013 18068682

[B22] Youngman P Perkins JB Losick R A novel method for the rapid cloning in Escherichia coli of Bacillus subtilis chromosomal DNA adjacent to Tn917 insertion Mol Gen Genet 1984 195 424 433 10.1007/BF00341443 6088944

[B23] Cutting S Vander Horn PB Harwood C, Cutting S Genetic analysis Molecular Biological Methods for Bacillus 1990 Chichester, UK, John Wiley and Sons 27 74

[B24] Kim H Hahn M Grabowski P McPherson D Otte MM Wang R Ferguson CC Eichenberger P Driks A The Bacillus subtilis spore coat protein interaction network Mol Microbiol 2006 59 487 502 10.1111/j.1365-2958.2005.04968.x 16390444

[B25] Zheng L Donovan WP Fitz-James PC Losick R Gene encoding a morphogenic protein required in the assembly of the outer coat of the Bacillus subtilis endospore Genes Dev 1988 2 1047 1054 10.1101/gad.2.8.1047 3139490

[B26] Chen G Driks A Tawfiq K Mallozzi M Patil S Bacillus anthracis and Bacillus subtilis spores surface properties and transport Colloids Surf B Biointerfaces 2010 76 512 518 10.1016/j.colsurfb.2009.12.012 20074921

[B27] Kazakov S Bonvouloir E Gazaryan I Physicochemical characterization of natural ionic microreservoirs: Bacillus subtilis dormant spores. J Phys Chem 2008 112 2233 2244 10.1021/jp077188u 18247596

[B28] Sambrook J Fritsch EF Maniatis T Molecular cloning, laboratory manual 1989 2 Cold Spring Harbor Laboratory Press, Cold Spring Harbor, NY, USA

[B29] Nicholson WL Setlow P Harwood C, Cutting S Sporulation, germination and out-growth Molecular biological methods for Bacillus John Wiley and Sons, Chichester, United Kingdom 391 450

[B30] Itaya M Kondo K Tanaka T A neomycin resistance cassette selectable in a single copy state in the Bacillus subtilis chromosome Nucleic Acid Res 1989 17 4410 10.1093/nar/17.11.4410 2500645 PMC317980

[B31] Ricca E Cutting S Losick R Characterization of bofA, a gene involved in inter-compartmental regulation of pro-K processing during sporulation in Bacillus subtilis J Bacteriol 1992 174 3177 3184 1577688 10.1128/jb.174.10.3177-3184.1992 PMC205984

[B32] Henriques A Beall BW Moran CP CotM of Bacillus subtilis, a member of the alpha-crystallin family of stress proteins, is induced during development and participates in spore outer coat formation J Bacteriol 1997 179 1887 1897 9068633 10.1128/jb.179.6.1887-1897.1997 PMC178911

[B33] Zilhao R Isticato R Martins LO Steil L Völker U Ricca E Moran CP Henriques AO Assembly and function of spore-coat associated transglutaminase of Bacillus subtilis J Bacteriol 2005 187 7753 7764 10.1128/JB.187.22.7753-7764.2005 16267299 PMC1280291

[B34] Isticato R Esposito G Zilhão R Nolasco S Cangiano G De Felice M Henriques AO Ricca E Assembly of Multiple CotC Forms into the Bacillus subtilis Spore Coat J Bacteriol 2004 186 1129 1135 10.1128/JB.186.4.1129-1135.2004 14762008 PMC344207

[B35] Sacco M Ricca E Losick R Cutting S An additional GerE-controlled gene encoding an abundant spore coat protein from Bacillus subtilis J Bacteriol 1995 177 372 377 7814326 10.1128/jb.177.2.372-377.1995 PMC176600

[B36] Naclerio G Baccigalupi L Zilhao R De Felice M Ricca E Bacillus subtilis spore coat assembly requires cotH gene expression J Bacteriol 1996 178 4375 4380 8755863 10.1128/jb.178.15.4375-4380.1996 PMC178202

